# Barriers to cardiac rehabilitation delivery in a low-resource setting from the perspective of healthcare administrators, rehabilitation providers, and cardiac patients

**DOI:** 10.1186/s12913-019-4463-9

**Published:** 2019-09-02

**Authors:** Thaianne Cavalcante Sérvio, Raquel Rodrigues Britto, Gabriela Lima de Melo Ghisi, Lilian Pinto da Silva, Luciana Duarte Novais Silva, Márcia Maria Oliveira Lima, Danielle Aparecida Gomes Pereira, Sherry L. Grace

**Affiliations:** 1 Rehabilitation Science Graduate Program, Universidade Federal de Minas Gerais, Belo Horizonte, Minas Gerais Brazil; 2 Cardiovascular Prevention and Rehabilitation Program, University Health Network, University of Toronto, Toronto, Canada; 3 College of Physical Therapy, Universidade Federal de Juiz de Fora, Juiz de Fora, Brazil; 4 Department of Applied Physical Therapy, Universidade Federal do Triângulo Mineiro, Uberaba, Brazil; 5 Department of Physical Therapy, Universidade Federal do Vale do Jequitinhonha e Mucuri, Diamantina, Brazil; 6 School of Kinesiology and Health Science, York University, Toronto, Canada

**Keywords:** Health care services, Cardiac rehabilitation, Cardiac care facilities, Attitude of health personnel

## Abstract

**Background:**

Despite clinical practice guideline recommendations that cardiovascular disease patients participate, cardiac rehabilitation (CR) programs are highly unavailable and underutilized. This is particularly true in low-resource settings, where the epidemic is at its’ worst. The reasons are complex, and include health system, program and patient-level barriers. This is the first study to assess barriers at all these levels concurrently, and to do so in a low-resource setting.

**Methods:**

In this cross-sectional study, data from three cohorts (healthcare administrators, CR coordinators and patients) were triangulated. Healthcare administrators from all institutions offering cardiac services, and providers from all CR programs in public and private institutions of Minas Gerais state, Brazil were invited to complete a questionnaire. Patients from a random subsample of 12 outpatient cardiac clinics and 11 CR programs in these institutions completed the CR Barriers Scale.

**Results:**

Thirty-two (35.2%) healthcare administrators, 16 (28.6%) CR providers and 805 cardiac patients (305 [37.9%] attending CR) consented to participate. Administrators recognized the importance of CR, but also the lack of resources to deliver it; CR providers noted referral is lacking. Patients who were not enrolled in CR reported significantly greater barriers related to comorbidities/functional status, perceived need, personal/family issues and access than enrollees, and enrollees reported travel/work conflicts as greater barriers than non-enrollees (all *p* < 0.01).

**Conclusions:**

The inter-relationship among barriers at each level is evident; without resources to offer more programs, there are no programs to which physicians can refer (and hence inform and encourage patients to attend), and patients will continue to have barriers related to distance, cost and transport. Advocacy for services is needed.

## Background

Cardiovascular diseases (CVD) are among the leading causes of morbidity and mortality worldwide, with over 80% of CVD deaths occurring in low- and middle-income countries (LMICs) [1]. In the middle-income country of Brazil for example, in 2013, 4.2% (6.1 million) of people 18 years of age or older had a diagnosis of some form of CVD [2].

Cardiac rehabilitation (CR) – a comprehensive outpatient program of secondary prevention and lifestyle changes [3] – can mitigate this burden. Robust evidence demonstrates positive effects of CR participation, including reductions of mortality up to 25% as well as decreases in hospitalizations [4]. Reduction in risk factors, as well as increase in quality of life and functional capacity are also reported in studies undertaken in LMICs [5, 6]. Dose-response associations are observed [7], hence it is not only important that patients enroll, but that they adhere and complete programs to achieve these benefits.

Despite consequent clinical practice guideline recommendations to refer CVD patients [8, 9], CR programs are highly unavailable and under-utilized, particularly in LMICs [10]. CR is only available in approximately 25% of LMICs [6, 10], with Brazil for example, having a density of 1 program per 4.9 million inhabitants [10]. The barriers are multifactorial, and include health system [11], referring physician, program and patient-level factors [12–16].

While complex, there are very few studies which consider these multi-level barriers concurrently [14, 17], and hence enable a fulsome understanding of the context of CR under-utilization, so that effective strategies to overcome them can be identified and implemented. There is even less data from LMICs [18] (only 13 studies identified, most not multi-level), which is a major omission considering [1] this is where the need for CR is greatest but availability is lowest, and [2] the context is considerably different than that of high-income countries (i.e., often private and public systems; low availability of primary healthcare). In South America there are only some discrete data on healthcare administrator perceptions of CR barriers [19], CR programs [20–22], as well as those among patients [23, 24]. Therefore, the aim of this study was to concurrently assess barriers to CR delivery at the healthcare system (including funding source), CR program, and patient (inclusive of barriers to not only enrolment, but also to adherence and completion of the program by enrollees) levels, in a low-resource context.

## Methods

### Design and procedure

Herein, data from cross-sectional surveys of three groups are presented, namely surveys of healthcare administrators, CR providers and patients. Integration across the three cohorts was undertaken in accordance with the principles outlined by Fetters et al. [25]. Data collection for all three samples occurred between February 2015 and May 2017. Approval was obtained from the Universidade Federal de Minas Gerais’ Ethics Committee (approval is at the state level; number 37156614.8.1001.5149).

To identify healthcare administrators, all public and private centers providing cardiac care were identified through the institutional lists of the State Department of Health, Minas Gerais, Brazil. The Brazilian national health system has a universal public health system and a supplementary health sector (private) [26]. Healthcare institutions with a cardiology unit (including inpatient and outpatient centers), as well as healthcare centers and private clinics providing any cardiac care were collated. As a check of completeness, an internet search was also performed using the following keywords: hospital, health center, cardiology, cardiac rehabilitation, the name of the state, and the name of each municipality in this state with more than 45,000 inhabitants.

CR centres across state were identified in a previous study through snowball sampling [21]. Additional sites were identified through a check of the institutions identified above, the Cardiorespiratory Physiotherapy and Physical Therapy in Intensive Care Association and key informants.

Each healthcare institution and CR program was contacted to request the email address of the appropriate staff member (i.e., person most responsible for administration of cardiac care) to direct the survey. An email was then sent to the indicated healthcare administrator or CR provider which explained the study, and contained a link to the applicable questionnaire (Survey Monkey**®**). Voluntary completion of the survey constituted informed consent. Telephone and e-mail reminders were sent after ten days without reply, with a maximum of five attempts. Identified staff reporting barriers to online completion were sent a printed copy of the survey via mail.

Cardiac patients were recruited from these public and private healthcare institutions across the state. A random sub-sample of six identified outpatient cardiac clinics (all private) and five hospitals (four public, 80.0%; to capture barriers to enrolling) as well as eleven CR programs (seven public, 63.6%; to capture barriers to program adherence and completion) was targeted. Participants completed a consent form and were asked about their barriers through a structured interview [27] performed in the waiting room before their medical appointment or CR session.

### Setting

The state of Minas Gerais is geographically expansive, which is reflective of the socio-economic variation that exists across the country. The majority of the population is served by publicly-funded health services; only the minority receive privately-funded healthcare [26]. Given the nature of care and access varies significantly by funding source, barriers were compared in publicly versus privately-funded institutions.

Similar to other settings, when referred, cardiac patients are generally referred to CR by a cardiologist, in the in- or out-patient settings. CR programs in the state generally offer structured exercise only (not comprehensive programs delivering all core components), delivered primarily by physiotherapists and physicians, 2 or 3 times per week and median duration of 15 weeks (Q25–75 = 12–16) [22]. There is no cost for patients to enroll in public CR services; for privately-funded programs, the cost varies between the equivalent of $100–300 USD per month [28], depending on whether the program is delivered in a group or individually.

### Participants

This study included three cohorts: healthcare administrators, CR providers, and cardiac patients. Healthcare administrators were defined as those directing or coordinating inpatient or non-CR outpatient cardiovascular health services. CR providers were defined as those in a regulated healthcare profession (e.g., physicians, physiotherapists, dietitians) providing CR care to patients. One most responsible cardiac healthcare administrator and CR coordinator designated per institution was invited to participate in the study, with no exclusion criteria.

Ischemic heart disease (+/− revascularization), heart failure or valve disease patients who were referred and enrolled in CR, and others who were not (actively identified in outpatient cardiology services associated with hospitals) were recruited. Patient inclusion criteria was eligibility to participate in CR based on CR Guidelines [29]. The exclusion criteria were: lack of language proficiency, and any visual, cognitive or psychiatric condition that would preclude the participant from understanding the survey.

While the entire population was invited to participate for the cardiology administrator and CR providers samples, a target sample size for patients was calculated, so that the study was powered to detect significant differences in CR barriers among enrollees versus non-enrollees. This was based on the equation for population-based studies by Van Belle and Fisher [30]. Assuming *p* = 0.46 (non-enrollee rate) [31] for non-participating cardiac patients, and 0.54 (enrollee rate) [31] for CR participants, a sample of 381 participants per group was sought (*N* = 762).

### Measures

#### CR delivery barriers from healthcare administrators’ perspective

Perceptions of cardiology healthcare administrators regarding CR have been previously systematically and quantitatively assessed in Latin American countries [19] and elsewhere [32]. The survey administered [32] was translated and adapted to the cultural context by a clinical researcher (R.R.B.) fluent in English and in Portuguese, and was pilot-tested prior to administration.

The survey began with items assessing who should fund CR programs, if there was team encouragement and systematization of referral to CR, whether the institution provided links to outpatient services for continuity of care, and whether there were sufficient resources to fund CR programs. This was followed by items assessing respondents’ knowledge (four items), perceptions (five items) and attitudes (fourteen items) regarding CR on a Likert-type scale. Knowledge items were scored from 1 = *poor* to 5 = *excellent*, perceptions items were scored from 1 = *not even considered* to 5 = *extremely important*, and attitude items from 1 = *strongly disagree* to 5 = *strongly agree* (some items were reverse-scored; see Table 2). Higher scores indicated more CR-positive perceptions / knowledge / attitudes. Finally, self-reported sociodemographic and occupational information was collected (e.g., professional position, years of service, sex, age, level of education, primary clinical area, type of healthcare institution [public or private], availability of CR in the institution [yes vs. no]).

#### Perceptions of CR providers about CR

A previously-validated questionnaire assessing the perceptions of CR providers regarding CR delivery barriers could not be identified in the literature, only one of referring providers [14, 15, 33] and a few barrier items in a larger survey of the nature of CR services more broadly [5]. Thus, an instrument was developed for this study, considering these previous measures and based on key literature [12, 13]. The instrument consisted of twelve items (see Table 3), with Likert-type response options ranging from 1 = *strongly disagree* to 5 = *strongly agree*; higher scores indicated more positive perceptions / attitudes. At the end of the instrument, there was an open-ended question where the respondent could state additional CR barriers beyond those listed. Again, the survey included self-report questions regarding sociodemographic and occupational characteristics.

#### Cardiac patients’ barriers

All cardiac patients were asked to self-report their sociodemographic characteristics (e.g., socio-economic level). Their clinical characteristics (e.g., cardiac diagnosis, cardiac history, risk factors) were extracted from medical charts. CR participants were questioned about time between referral and initiation of the program, as well as the number of absences from the program and reasons for these absences, when this information was not available in medical charts.

All patients were invited to respond to the Cardiac Rehabilitation Barriers Scale (CRBS; verbal administration) [27]. This scale assesses patient’s perceptions of the degree to which patient, provider, and health system-level barriers affect their CR enrollment and participation. Regardless of CR referral or enrollment, participants are asked to rate their level of agreement with each of the 21 barrier statements, and report additional CR barriers beyond those in open-ended fashion. Items were rated on a 5-point Likert-type scale that ranged from 1 = *strongly disagree* to 5 = *strongly agree*. Higher scores indicated greater barriers to enrolment or adherence / completion of CR as applicable.

The CRBS was developed and psychometrically-validated by Shanmugasegaram and colleagues in English [31]. It was later translated, culturally-adapted and psychometrically-validated in Brazilian-Portuguese [27]. This version consists of five subscales: comorbidities / functional status, perceived need, personal/family issues, travel/work/time conflicts and access (see Table 4).

### Statistical analyses

Statistical analysis was performed using the IBM software Statistical Package for Social Sciences (SPSS) version 21.0. First, descriptive analyses on data from all three cohorts were performed. To compare patient barriers in CR participants versus non-participants and also to explore differences in barriers by institutional funding source (public vs. private), the Mann-Whitney U test was used. A value of *p* < .01 was used to denote significance given the multiple comparisons. Finally, data from the three cohorts were integrated / triangulated [25] to derive conclusions and consider implications.

## Results

### Respondents characteristics

Ninety-one institutions providing cardiology services were identified across the state of Minas Gerais; of these, 47 (51.6%) were publicly-funded. Healthcare administrators from 32 (35.2%) institutions responded: 24 (75.0%) from public (14 hospitals, and 10 outpatient clinics) and eight (25.0%) from private (5 hospitals, and 3 outpatient clinics) institutions. Only five (15.6%) respondents opted for the mailed printed survey. Of the 19 hospitals, 16 (84.2%) had intensive care, and five (31.2%) had a CR program. The characteristics of the healthcare administrators are shown in Table 1.

**Table 1:** Characteristics of healthcare administrators, cardiac rehabilitation providers, and cardiac patients

Characteristics	N (%)
HEALTHCARE ADMINISTRATORS	*N* = 32
Sex	
Male	20 (62.5%)
Highest Educational Attainment	
Post-Secondary	20 (62.5%)
Post-Graduate	12 (37.5%)
Professional position	
Clinical Director	13 (40.6%)
Manager	9 (28.1%)
General Director	2 (6.3%)
Cardiology Coordinator	2 (6.3%)
Other	6 (18.7%)
Cardiac rehabilitation providers	*N* = 16
Sex	
Female	10 (61.5%)
Highest Educational Attainment	
Post-Graduate	16 (100.0%)
Healthcare Profession	
Physiotherapist	7 (43.7%)
Physician	3 (18.7%)
Exercise specialist	3 (18.7%)
Dietitian	1 (6.3%)
Nurse	1 (6.3%)
Other	1 (6.3%)
CARDIAC PATIENTS	*N* = 805
Sociodemographic	
Age, years (mean ± SD)	62.85 ± 12.42
Sex, n (%)	
Male	442 (54.9%)
Marital status	
Single	121 (15.0%)
Married	488 (60.6%)
Divorced	79 (9.8%)
Widowed	117 (14.5%)
Highest Educational Level	
Elementary School	447 (55.7%)
High School	198 (24.7%)
Post-Secondary	138 (17.2%)
Post-Graduate	20 (2.5%)
Clinical^a^	
Cardiac History	
Coronary Artery Disease	500 (61.4%)
Myocardial Infarction	337 (41.4%)
Percutaneous Coronary Intervention	267 (32.8%)
Heart Failure	92 (11.3%)
Arrhythmia	181 (22.5%)
Valve Disorder	83 (10.3%)
Risk Factors	
Hypertension	646 (79.4%)
Dyslipidemia	410 (50.4%)
Smoking history	410 (50.4%)
Diabetes	233 (28.6%)
Cardiac rehabilitation (enrollees)	*n* = 305
Wait time in months (mean ± SD)	4.03 ± 5.74
Number of missed sessions in last month (mean ± SD)	1.60 ± 1.82

*SD* standard deviation. ^a^ Extracted from medical charts. All other data are self-reported

Forty-one CR programs were identified, of which nine (21.9%) were publicly-funded. Responses from providers at 16 (39.0%) programs were received. Their characteristics are shown in Table 1.

The sample of cardiac participants consisted of 805 respondents. Their sociodemographic and clinical characteristics are shown in Table 1. In total, 495 (61.5%) participants were from public, and 310 (38.5%) participants were from private institutions. Overall, 305 (37.9%) patients were enrolled in CR; sex and age for enrollees and non-enrollees are shown in Table 4.

### Perceptions of healthcare administrators

The majority of respondents (*n* = 23, 71.9%) stated that CR programs should be funded by the Ministry of Health and 15 (46.9%) by private health plans. Also, most of the healthcare administrators (*n* = 21; 65.6%) considered CR as a good use of public healthcare resources.

Seventeen (53.1%) respondents agreed that acute care institutions are responsible for providing patient connections to outpatient services for continuity of care. Nine (28.1%) encouraged physicians and residents to refer participants to CR, but without systematization. Seven (21.9%) institutions had systematic CR referral. In five (15.6%), referral was hardly or never discussed at meetings.

All (100.0%) respondents indicated their institutions did not have sufficient resources for CR, and lacked capacity to provide care to referred patients, but the healthcare administrators affirmed that they perceived their institution would provide more support if more financial resources were available (again 100.0%).

Table 2 shows mean scores on the knowledge, perceptions and attitudinal items. Overall, the healthcare administrators had satisfactory to good knowledge about CR. Their perceptions towards CR were very positive, and attitudes moderately positive.

**Table 2:** Healthcare administrators’ knowledge, perceptions and attitudes regarding cardiac rehabilitation, *N* = 32

Item	mean ± SD
KNOWLEDGE^a^	
My knowledge of what CR entails	2.75 ± 1.34
Rates of participation in CR at the institution where I am employed	2.09 ± 1.11
The location of the nearest CR program	2.00 ± 1.29
Level of knowledge about CR of my colleagues	1.71 ± 0.85
PERCEPTIONS^b^	
The importance of CR for outpatient care	4.37 ± 0.55
The role of CR access programs in reducing patient length of stay	4.18 ± 0.64
The role of CR programs in reducing re-admissions	4.15 ± 0.76
The importance of care of patients with other vascular conditions in CR	4.00 ± 0.76
Perceptions of your institution about the importance of CR	3.81 ± 0.85
ATTITUDES^c^	
CR programs provide benefits beyond what primary care providers can offer	4.28 ± 0.72
CR programs promote sustainedbehavioral changes that improve patient outcomes	4.09 ± 0.92
It is likely that government funding for CR programs will be sustained over time	4.06 ± 0.80
It is the hospital’s responsibility to provide all eligible inpatients with the information they need to begin CR	3.87 ± 1.00
The government should provide more funding for CR	3.87 ± 0.65
Government ministry funding models are a financial disincentive to CR provision^d^	3.68 ± 1.09
Patients and their families should be responsible for their own health behavior changes and risk reduction self-management posthospitalization^d^	3.46 ± 1.31
We do not have enough space to run a CR program at my institution^d^	3.40 ± 1.26
The closest available CR program is of good quality	3.15 ± 0.84
CR services are generally one of the first programs to be cut back when we make budget reductions^d^	2.65 ± 1.00
Scarce healthcare money should not be spent on outpatient care at the expense of acute care^d^	2.25 ± 1.13
Health care providers on the cardiac floor have other more important clinical duties than to refer patients to CR^d^	1.90 ± 0.77
I am skeptical about the benefits of CR programs^d^	1.84 ± 0.76
Government health insurance should not cover CR services for cardiac patients post-hospitalization^d^	1.56 ± 0.50

*CR* cardiac rehabilitation, *SD* standard deviation

^a^: Items were scored on a scale from 1 “poor” to 5 “excellent”

^b^: Items were scored on a scale from 1 “not even considered” to 5 “extremely important”

^c^: Items were scores on a scale from 1 “strongly disagree” to 5 “strongly agree”

^d^: These items were displayed in reverse-scored

### Perceptions of CR providers

The perceptions of CR providers regarding CR delivery are shown in Table 3. It is the perception of CR staff that referring physicians are not sufficiently aware of the benefits of CR and do not refer. Respondents were highly supportive of providing fully comprehensive CR.

**Table 3:** Perceptions of Cardiac Rehabilitation Staff on Delivery (*N* = 16)

Item	mean ± SD
FACILITATORS AND BARRIERS^a^	
CR participants understand the benefits of joining the program	4.50 ± 0.51
Most physicians do not refer patients to CR	4.31 ± 0.60
Most physicians are unaware of the benefits of CR	4.06 ± 0.68
The rate of absenteeism in my program is very low	3.75 ± 1.00
Delivering hybrid CR^c^ could increase participation by patients	3.62 ± 1.02
Participants enrolled in CR have difficulty staying in the program	3.50 ± 1.03
Many patients are referred by doctors, but choose not to participate	3.12 ± 1.14
Our program could serve a larger number of participants, but there is no demand	2.93 ± 1.80
DELIVERY OF COMPREHENSIVE CR^b^	
Access to optimal medical therapy and reinforcement of the need to adhere to pharmacological treatments	4.87 ± 0.34
The assessment and control of patient’s blood pressure, glucose and lipids	4.81 ± 0.40
The inclusion of a comprehensive educational component within CR	4.75 ± 0.44
Adequate physical space and resources to offer comprehensive CR	4.43 ± 0.51

*CR* cardiac rehabilitation, *SD* standard deviation

^a^Items were scores on a scale from 1 “strongly disagree” to 5 “strongly agree”

^b^ Items were scored on a scale from 1 “not even considered” to 5 “extremely important”

^c^ supervised and unsupervised exercise, thus requiring fewer on-site visit

CR providers reported several additional barriers such as: high cost of CR programs for patients, lack of government initiative to create more CR programs, low educational level of patients (elementary school), lack of patient motivation to change habits, and lack of knowledge about CR by the non-medical professionals (e.g., nurses, dietitians).

### Perceptions of cardiac patients

The CRBS item and subscales scores are shown in Table 4. The greatest barriers were related to lack of awareness and encouragement by physicians. Cardiac patients reported some additional barriers, which related to distance and transportation (items already assessed in CRBS).

**Table 4:** Mean Cardiac Rehabilitation Barrier Scale scores by funding source and CR participation status

	Total	Institution Funding source		CR participation status	
	*N* = 805	Public *n* = 495 (61.5%)	Private *n* = 310 (38.5%)	Enrolled *n* = 305 (37.9%)	Not *n* = 500 (62.1%)
Sex					
Male (%)		297 (60%)	139 (45%)	191 (63%)	245 (50%)
Age (mean ± SD)		60.8 ± 11.1	65.1 ± 13.7**	65.4 ± 11.4**	61.2 ± 12.7
CRBS item (number) / subscale ^a^ mean ± SD					
I didn’t know about CR [5]	3.17 ± 1.54	3.14 ± 1.56	3.22 ± 1.52	1.97 ± 1.03	3.90 ± 1.34***
My doctor did not feel it was necessary [16]	2.63 ± 1.43	2.63 ± 1.42	2.63 ± 1.46	1.65 ± 0.68	3.23 ± 1.45***
Distance [1]	2.43 ± 1.41	2.60 ± 1.45***	2.16 ± 1.31	2.05 ± 1.09	2.66 ± 1.54***
Cost [2]	2.42 ± 1.39	2.62 ± 1.45***	2.08 ± 1.21	2.09 ± 1.09	2.60 ± 1.50**
Transportation problems [3]	2.26 ± 1.31	2.45 ± 1.38***	1.93 ± 1.12	2.02 ± 1.00	2.40 ± 1.45
I don’t need CR [6]	2.25 ± 1.29	2.08 ± 1.14	2.54 ± 1.45***	1.63 ± 0.66	2.64 ± 1.43***
Travel [10]	2.13 ± 1.48	1.97 ± 1.10	2.40 ± 1.92**	2.64 ± 1.80***	1.83 ± 1.15
Other health problems [14]	2.13 ± 1.25	2.11 ± 1.24	2.13 ± 1.24	2.31 ± 1.31**	2.00 ± 1.19
I find exercise tiring or painful [9]	2.07 ± 1.14	2.09 ± 1.13	2.03 ± 1.14	1.78 ± 0.81	2.24 ± 1.26***
I can manage my heart problem on my own [18]	2.04 ± 1.13	2.03 ± 1.09	2.06 ± 1.19	1.82 ± 0.87	2.18 ± 1.24**
I already exercise at home, or in my community [7]	2.01 ± 1.13	1.97 ± 1.05	2.09 ± 1.24	1.71 ± 0.69	2.21 ± 1.29***
Many people with heart problems don’t go, and they are fine [17]	2.01 ± 1.07	1.99 ± 1.05	2.04 ± 1.11	1.88 ± 0.88	2.09 ± 1.17
Family responsibilities [4]	2.00 ± 1.36	2.04 ± 1.52	1.95 ± 1.08	2.03 ± 1.04	1.99 ± 1.53
I don’t have the energy [13]	1.99 ± 1.14	1.95 ± 1.11	2.05 ± 1.17	1.73 ± 0.82	2.14 ± 1.27**
Time constraints [11]	1.98 ± 1.11	1.96 ± 1.07	2.02 ± 1.16	1.68 ± 0.64	2.16 ± 1.28**
Work responsibilities [12]	1.94 ± 1.13	1.92 ± 1.11	2.00 ± 1.16	1.88 ± 0.94	2.00 ± 1.23
Severe weather [8]	1.90 ± 1.03	1.96 ± 1.07	1.82 ± 0.95	1.90 ± 0.95	1.91 ± 1.08
I prefer to take care of my health alone, not in a group [21]	1.86 ± 1.05	1.76 ± 0.96	2.01 ± 1.15**	1.68 ± 0.71	1.96 ± 1.19
It took too long to get referred into the program [20]	1.69 ± 0.82	1.76 ± 0.89	1.59 ± 0.68	1.87 ± 0.89***	1.58 ± 0.75
I am too old [15]	1.65 ± 0.86	1.65 ± 0.88	1.67 ± 0.82	1.59 ± 0.71	1.70 ± 0.94
I think I was referred, but the rehab prog didn’t contact me [19]	1.62 ± 0.73	1.67 ± 0.79	1.53 ± 059	1.71 ± 0.70***	1.56 ± 0.73
SUBSCALES					
Subscale 1 Comorbidities / functional status	1.98 ± 0.74	1.97 ± 0.72	1.99 ± 0.77	1.84 ± 0.58	2.07 ± 0.81***
Subscale 2 Lack of perceived need	2.44 ± 0.84	2.43 ± 0.88	2.45 ± 0.77	1.80 ± 0.54	2.83 ± 0.75***
Subscale 3 Personal / family issues	2.04 ± 0.80	2.04 ± 0.83	2.06 ± 0.77	1.85 ± 0.61	2.17 ± 0.88***
Subscale 4 Travel / work conflicts	2.06 ± 1.03	1.96 ± 0.89	2.23 ± 1.20***	2.25 ± 1.10***	1.95 ± 0.96
Subscale 5 Access	2.03 ± 0.79	2.16 ± 0.81***	1.84 ± 0.70	1.93 ± 0.67	2.10 ± 0.84**
Total Score	2.12 ± 0.57	2.12 ± 0.58	2.11 ± 0.54	1.89 ± 0.51	2.26 ± 0.55***

*CR* cardiac rehabilitation, *CRBS* cardiac rehabilitation barriers scale, *SD* standard deviation

Differences by funding source and CR participation status: Mann-Whitney test; **p < .01; ***p < .001

^a^Items were scored on a scale from 1 (“strongly disagree”) to 5 (“strongly agree”). Higher scores indicate greater barriers to participation or adherence to CR programs

Differences in barriers by program funding source and CR enrolment status are also shown in Table 4. Respondents from public institutions considered distance, cost and transportation to be significantly greater barriers to CR participation than those from private institutions (and correspondingly the access subscale was significantly greater among patients from public institutions); Respondents from private institutions considered the following factors to be significantly greater barriers to CR participation than those from public institutions: lack of perceived need, travel (and correspondingly the travel/work conflicts subscale was significantly greater among patients from public institutions) and preferring to manage their chronic condition independently.

Cardiac patients who did not go to CR considered the following factors to be significantly greater barriers to CR participation than those who did: lack of awareness of CR, lack of physician encouragement, distance, cost, lack of perceived need, finding exercise tiring or painful, preferring to self-manage their chronic condition, already exercising at home or in their community, not having the energy, and time constraints (and correspondingly 4 of the 5 subscales were significantly greater among non-enrollees); CR participants endorsed travel, comorbidities, wait times, and lack of program follow-up as a barrier to a significantly greater degree than did non-participants (the travel/work conflict subscale was significantly greater in this group; Table 4).

## Discussion

In this first study to concurrently consider CR barriers in a low-resource setting from the perspective of healthcare administrators, CR providers, and cardiac patients, lack of resources and funding, lack of referral / physician encouragement, lack of patient awareness, and poor access for patients (i.e., distance, cost, transportation) were the main barriers identified. There is a clear incongruity between the recognition of the importance of CR and its effective implementation and use by healthcare administrators, and the low supply of CR programs and lack of resources to deliver services. Lack of referral is a key theme across all levels. The inter-relationship between the barriers at each level is evident – specifically without resources to offer programs, there are no programs to which physicians can refer (and hence inform and encourage patients to attend), and patients will continue to have barriers related to distance, cost and transport.

The healthcare administrators had low to moderate knowledge and attitudes about CR, but very positive perceptions. When compared to administrators in high-income countries [32], their knowledge is much lower (means all above 3 in Canada), and their perceptions and attitudes are somewhat less positive. There was a notable discrepancy with regard to CR space, with Canadian administrators rating this as much less of an issue. In a survey of CR programs in Latin America [20, 21] and the Arab world [10] (of which many are LMIC), lack of space was also among the greatest barriers to CR provision.

Issues identified by programs included lack of physician referral, likely caused by the perceived lack of awareness of CR among physicians. They also reported lack of programs as a major issue; Indeed the low availability of CR programs in Minas Gerais has been previously established [22]. In a review of national/regional surveys of CR 4barriers were human resources, financial resources and space. In a review on CR barriers in LMICs specifically [18], the most frequently-reported barriers were lack of personnel and resources, as well as profitability.

With regard to patients, the major barriers among non-enrollees were lack of awareness and physician encouragement and barriers to program adherence among enrollees were travel, comorbidities, cost, distance, and family responsibilities. It is not appropriate to compare CRBS scores between studies except where data are shown by CR enrolment status (given the major differences in barriers, the proportion of enrollees and non-enrollees in the cohort would impact mean values), but scores were reported by enrolment in a sample of Brazilian cardiac patients [23]. The findings herein are fairly consistent with that study, showing that the greatest barriers among non-enrollees were distance, lack of awareness and lack of encouragement, and among enrollees were travel and cost. In the review of CR delivery around the world [34], patient-related barriers in LMICs were also lack of awareness, cost, transportation and time constraints.

Multi-level strategies to overcome these barriers in LMICs are forwarded in the review by Ragupathi et al. [18]. In relation to the systems factors, for example, we can align incentives with service delivery and improve revenue streams, as well as CR delivery in community health service centers [34], exploiting existing physical infrastructure (e.g., community exercise centers). We need more programs before we can promote and automate CR referral by physicians [35]. CR programs themselves should be comprehensive, but simplified [35], low-cost [28] models have been forwarded, which should exploit unsupervised delivery modalities (e.g., smartphones) [36]. Finally, evidence-based strategies that motivate the participation and adherence of patients such as counseling by clinicians should be applied [37]. Studies evaluating the effect of such strategies in LMICs are scarce, and clearly this represents an important direction for future research.

This study has several limitations that need to be considered. First, as there is no official directory of cardiac care centers and CR programs in the state evaluated, it is possible that all programs were not identified (and in particular smaller ones), which may introduce selection bias. Second, the response rate was low for the healthcare administrators and CR providers, mainly in privately-funded services, suggesting that the results herein may be less representative of barriers in private care. Moreover, the response rate in patients was not captured, and hence there could be selection bias (e.g., higher socioeconomic status, more motivated patients represented in the sample than the average cardiac patient).

Third, the design was cross-sectional and therefore no causal conclusions should be drawn. Fourth, the cohorts were recruited from only one country state, thus results might not be generalizable to other states in Brazil, or to other low-resource settings more broadly. Fifth, the healthcare administrator survey was not professionally translated, nor was a formal process of cross-cultural adaptation applied. Therefore, the validity and reliability of that assessment is unknown. Sixth, CR-referring physicians were not directly surveyed; consideration of referral barriers were made indirectly through the CR provider and patient surveys. However, findings were consistent with other research. Finally, there were age differences in patients enrolling vs not in CR, which may be related to the barriers identified.

## Conclusions

While CR is greatly needed to mitigate the epidemic of CVD in LMICs, there remain major barriers at the system, provider, program and patient levels that must be addressed to ensure all indicated patients access programs. While perceptions of CR are very positive, knowledge among healthcare administrators and referring physicians is low, and all sites considered CR to be insufficiently resourced. Patients reported barriers related to distance, cost and transportation problems. Lack of referral was again identified as a major barrier to CR use, however we must first develop programs with sufficient capacity to which patients can be referred.

## Data Availability

All data are available from authors upon request.

## Abbreviations

CR: Cardiac rehabilitation
CRBS: Cardiac Rehabilitation Barriers Scale
CVD: Cardiovascular diseases
LMICs: Low- and middle-income countries
USD: United States Dollar
